# Practical Anatomy; Geneva College, and the University of the City of New-York

**Published:** 1847-01

**Authors:** 


*Practical Anatomy; Geneva College, and the University of the City of New-York.*—We publish the following printed circular by Prof. Webster, as an act of justice, not only to that gentleman, and his colleagues, whose action on the *subjects* involved, (we are half disposed to have this considered a *pun*,) will, we do not doubt, be properly appreciated by the profession; but, also, to the medical faculty of the University of the City of New-York, who have, as will be perceived, retracted statements made in their annual circular, upon which comments were made in a former number of this journal.—*Editor Buff. Jour.*

*To the Medical Profession of the City of New-York.*

The subscriber deems an explanation due to the Profession of New-York in relation to a matter that has recently caused some remark. The facts, without comment, are all that is deemed necessary, for the present, to state in relation to the difficulties between Geneva Medical College, and the Medical Department of the University of the City of New-York, in Broadway, above Bleecker-street.

"At a meeting of the Medical Faculty of Geneva College, held in the College Buildings, Geneva, on Saturday, October 10th, 1846, the following Premable and Resolutions were adopted:

" Whereas, In the circular published by the Medical Department of the University of New-York, during the Summer of 1846, the following passage occurs:

" 'The facilities which the city of New-York presents for the study of Practical Anatomy, are well known throughout the breadth of the land. Her immense population allows a large surplus of the *materiel;* and from this *surplus*, Schools of Medicine, in almost all parts of the country, have mainly drawn their supplies, thus instituting a traffic most dangerous to the interests of Practical Anatomy. The attention of the municipal authorities has been drawn to the subject, and hereafter, it is understood, the *trade* will be at an end, so far as the city of New-York is concerned. The arrangements for providing the University with the necessary *materiel* are perfect, and the students enjoy opportunities for the pursuit of Practical Anatomy second only to those offered by the city of Paris. The *materiel* is not only abundant, but it is likewise extremely *cheap*, and is always furnished to the classes promptly and without fail, as often as may be required.'

"And Whereas the above quotation would seem to imply that the Mayor, and members of the Common Council of the City of New-York, are parties to the procurement of the dead bodies of the poor of that city, for the purpose of dissection in the University of New-York, and that their influence is also used to inflict injury upon "Schools of Medicine, in almost all parts of the country," by attempting to convey the impression that Medical Schools abroad depend exclusively upon "supplies from New-York:"—Therefore—

Resolved, That as the laws of this State protect alike the dead bodies of the poor as well as the rich, therefore the Professors of New-York have no more right to them than those of any other Institution, or individual, in or out of the State, and are subject to the same penalties for their infringement."

Here follows a series of Resolutions expressing our disbelief in any participation of the "Municipal Authorities," and urging in strong terms our condemnation of what we deemed an assumption of power and unprofessional conduct on the part of the Medical Faculty of the University of New-York.

After a correspondence with that Faculty, it is but just to them to say that the following communication, dated November 9th, 1846, is perfectly satisfactory.

*University, New-York, Nov. 9th,* 1846.

To his Honor, the Mayor of the City of New-York.

Sir:—The Medical Faculty of the University of New-York, having learned with regret that a misapprehension exists in reference to a paragraph contained in their last circular on the subject of Practical Anatomy, deem it due to themselves and to the Municipal Authorities of the City of New-York, to disclaim any intention to hold out in that circular the idea that those authorities, either directly or indirectly, sanction the furnishing to them any facilities whatever for the supply of *material* for the purpose of anatomical instruction.

Having further learned that the Medical Faculty of Geneva College have taken objection to the paragraph referred to, conceiving that it was intended to convey the idea that that College was deficient in the supply of *material* for dissection; the Faculty of that University disclaim any such intentien, either as respects that or any other medical institution; and it affords them pleasure to be informed by the Faculty of Geneva College that the supply of that institution from "legitimate sources," is ample.

The Faculty of Geneva College, without any previous correspondence with the University Faculty, having published resolutions of the most offensive character, to which we would respectfully refer you, we conceive, (and we believe we shall be sustained by your honor,) that it is impossible for us to make any explanation to Geneva College until those resolutions are withdrawn. Then, it will give us pleasure to make the same explanation to them, that we now do to your honor.

By order of the Faculty:

(Signed,) JNO. W. DRAPER,

*Secretary of the Faculty.*

I do hereby certify the above to be a true copy.

A. H. MICKLE, MAYOR.

*New-York, 14th November,* 1846.

In regard to the last paragraph of the above letter, the Faculty of Geneva Medical College have only to say that, having expressed nothing in the resolutions which they can properly retract, they are quite willing to leave the difference of opinion between them and the University on that subject, to the candid judgment of the Profession.

This brief exposition, it is hoped, will explain the nature of the difficulty, and prevent hereafter a repetition of aggressions upon the rights and interests "of Schools of Medicine in almost all parts of the country."

JAS. WEBSTER,

*Prof. of Anatomy, Geneva Med. College.*

CARLTON HOUSE,

*New-York, Nov. 14th,* 1846.

